# Effect of body condition score loss during the transition period on metabolism, milk yield and health in Holstein cows

**DOI:** 10.2478/jvetres-2025-0004

**Published:** 2025-02-25

**Authors:** Rui Sun, Xuejie Jiang, Yu Hao, Ying Li, Yunlong Bai, Cheng Xia, Yuxi Song

**Affiliations:** College of Animal Science and Veterinary Medicine, Heilongjiang Bayi Agricultural University, Daqing 163319, China

**Keywords:** dairy cows, body condition score, milk yield, metabolism, disease

## Abstract

**Introduction:**

This study aimed to investigate the impact of perinatal body condition score (BCS) and its subsequent loss on postpartum performance and health outcomes in dairy cattle.

**Material and Methods:**

A total of 156 cows were randomly selected, and blood samples were collected at –21, 0, 7, 14, 21, 28 and 50 days relative to calving. Milk yield and disease incidence in dairy cows were recorded after calving. These cows were subsequently categorised into three groups based on BCS loss during the transition period: a no-BCS-loss (maintained BCS) group (M, 0 < BCS loss ≤ 0.25), low-BCS-loss group (L, 0.25 < BCS loss ≤ 0.5), and high-BCS-loss group (H, BCS loss > 0.5).

**Results:**

All groups experienced a decline in BCS from 21 days prepartum through 50 days postpartum (P-value < 0.01). Cows in the H group had the highest levels of non-esterified fatty acids, beta-hydroxybutyrate, total cholesterol, aspartate aminotransferase, albumin, malondialdehyde and leptin (P-value < 0.05). Concomitantly, total antioxidant capacity, as well as the levels of insulin and glucose, were the lowest in group H (P-value < 0.05). Plasma concentrations of Ca, P, Mg and K, urea nitrogen and total bilirubin were not significantly influenced by BCS loss (P-value > 0.05). Cows in the M group were less likely to develop ketosis, mastitis, retained placenta, displaced abomasum and metritis than those in the H group, and cows in the H group produced the lowest milk yields (P-value < 0.05).

**Conclusion:**

These observations collectively indicate that BCS loss is associated with measurable changes in energy balance, liver function, oxidative stress, daily milk production and disease incidence during the transition period.

## Introduction

During the transition period, dairy cows undergo significant physiological changes (37). There is a pressing need to optimise the management and monitoring of the energy reserves of dairy cows, as fluctuations in them can significantly impact cows’ performance and health, particularly when their energy needs exceed their energy intake levels. Body condition score (BCS), a subjective measurement system frequently used on dairy farms, is utilised to assess body fat reserves (40).

Prior studies indicated a correlation between the BCS of cows and the amount of subcutaneous fat, which serves as their primary energy source (19). Overall, the BCS of dairy cows reflects nutritional status, predicts milk yield and assists in formulating feeding strategies (17). Effective management of body conditions is essential for maximising reproductive and lactation performance, minimising the risk of negative energy balance (NEB), mitigating vulnerability to diseases and enhancing profitability in dairy farming. Unmanaged, alterations in body condition can impact the metabolic function of dairy cows, with BCS loss predominantly affecting serum metabolite levels (5, 9, 15). Excessive postpartum BCS loss may lead to NEB, which can cause increased oxidative stress and metabolic disturbances (6, 45).

Excessive postpartum BCS loss is associated with elevated levels of blood non-esterified fatty acids (NEFA) and beta-hydroxybutyrate (BHB), as well as decreased levels of glucose and total cholesterol (TC), signalling a state of NEB (14, 43). This NEB state arises from an increased demand for lactose synthesis, leading to elevated leptin levels and reduced insulin concentrations (22, 33). The development of NEB in cows can impair liver functions, as evidenced by elevated levels of aspartate aminotransferase (AST) and albumin (ALB) (29, 30). Furthermore, impaired liver function during NEB inhibits the metabolic clearance of urea (8). Malondialdehyde (MDA) in dairy cows results from the oxidative degradation of lipids, while total antioxidant capacity (T-AOC) serves as an evaluation index of comprehensive antioxidant capacity (31, 44). An earlier study established that high-BCS cows exhibited the highest levels of MDA, with its levels increasing on the day of calving and subsequently decreasing postpartum (46). Conversely, T-AOC levels displayed an opposite trend (2). Macromineral levels, including those of calcium, phosphorus, and magnesium, are tightly regulated in cows, and their regulation plays a vital role in maintaining overall body homeostasis (27).

Increased BCS in dairy cows is a predisposing factor for ketosis and is strongly correlated with metabolic diseases (28). Cows with higher BCS at the end of lactation tend to eat less during the dry period, leading to potential loss of BCS and an increased risk of uterine diseases (10, 11). Additionally, a correlation has been observed between BCS and the incidence of milk fever in dairy cows (41). Fluctuations in BCS during the transition period can also impact the occurrence of lactation-related issues in dairy cows (1).

This study aimed to investigate the impact of perinatal BCS and its subsequent decline on postpartum performance and health outcomes in dairy cattle, with a focus on reducing the incidence of postpartum diseases, optimising production capabilities, and enhancing the overall welfare, health and productivity of dairy cows in intensive cattle farms located in Heilongjiang Province, China. The factors reflected in BCS collectively play a pivotal role in metabolic health and can potentially compromise the performance of dairy cattle.

## Material and Methods

All animal experiments were approved by the Institutional Animal Care and Use Committee of Heilongjiang Bayi Agricultural University (Daqing, China) (protocol code DWKJXY2024007, approval date 4 January 2024).

### Animals

This prospective observational experiment was conducted on a commercial farm in Heilongjiang Province from January 2024 to April 2024. The experiment was conducted from 21 d prepartum to 50 d postpartum. One hundred and fifty-six healthy primi- or multiparous Holstein dairy cows (aged 2–4 years, parity 1–3) were stratified into groups based on their perinatal BCS loss (*i.e*. BCS at 21 d postpartum minus BCS at 21 d prepartum): a no-BCS-loss (maintained BCS) group (M, n = 40, 0 < BCS loss ≤ 0.25); a lost-BCS group (L, n = 52, 0.25 < BCS loss ≤ 0.5); and a high-BCS-loss group (H, n = 64, BCS loss > 0.5). The subject animals were housed in free-stall barns with continuous freshwater access and were milked three times daily. According to the 2001 standards of the US National Research Council, total mixed rations were formulated for early lactation (25). Specifically, the total mixed rations consisted of 4.5 kg of concentrate, 12 kg of silage, 3 kg of oat grass, 0.5 kg of cornflakes, 0.04 kg of yeast and 5 kg of water. Feed analysis showed the roughage content to be 59.45%, the crude protein content to be 18.72%, crude fat to be 2.02%, natural detergent fibre to be 41.08% and acid detergent fibre to be 22.59%fibre.

### Data collection

Age, parity and milk yield were documented using dedicated software (Afifarm, Afimilk, Kibbutz Afikim, Israel). At –21, 0, 7, 14, 21, 28 and 50 d relative to calving, two qualified field veterinarians determined the BCS using a five-point scale with 0.25-unit intervals (13). Body condition score loss was calculated as the difference between the BCS at 21 d prepartum and the BCS at 21 d postpartum.

### Blood collection

At –21, 0, 7, 14, 21, 28 and 50 d relative to calving, 10 mL of blood was collected from the tail vein and placed in an anticoagulant tube (Becton Dickinson, Franklin Lakes, NJ, USA) containing sodium heparin before milking and while cows were fasting in the morning. Following centrifugation at 1,500 × *g* for 5 min, the supernatants from centrifuged anticoagulated blood were placed in 1.5 mL Eppendorf tubes. After centrifugation at 12,000 × *g* for 5 min, 500 μL of the supernatant was transferred to 1.5 mL Eppendorf tubes and stored at –80°C for biochemical analysis and radioimmunoassay.

### Plasma detection

A Mindray BS-830S fully automatic biochemistry analyser (Mindray Biomedical Electronics Co., Shenzhen, China) at Heilongjiang Bayi Agricultural University was employed to measure the plasma levels of BHB, NEFA, glucose, TC, urea nitrogen (UN), AST, ALB, total bilirubin (TBIL), Ca, P, Mg and K using commercial biochemical assay kits (Mindray Biomedical Electronics Co., Shenzhen, China) following the manufacturer’s protocols. Another commercial assay kit (Nanjing Jiancheng Bioengineering Institute, Nanjing, China) was used to determine plasma T-AOC and MDA levels.

The serum concentrations of insulin and leptin were determined with commercial kits (Xinfan Biotechnology Co., Shanghai, China) and validated *via* radioimmunoassay procedures according to the instructions of the kits. The sensitivity of the insulin assay was 2 IU/mL, and that of the leptin assay was 0.1 ng/mL. Inter- and intra-assay variances were <10% and <15%, respectively.

### Diagnosis of health conditions

As reported from previous studies (4, 12, 39, 41), cows manifesting decreased appetite and altered patterns of milk yield were subjected to urine testing for ketone bodies (Keto-Stix; Bayer Diagnostics, Tarrytown, NY, USA), and those that tested at or above moderate levels were diagnosed with ketosis. Clinical mastitis was identified by herd personnel, with abnormal milk or inflammation of the quarters serving as diagnostic signs. During milking, the herd personnel inspected three rounds of milk for abnormalities. Milk fever is defined as a disorder of a prostrated cow with minimal rumen contractions which responds within 30 min to intravenous calcium. The diagnosis of displaced abomasum involved identifying a metallic (ping) sound upon percussion auscultation of the abdomen between the 4^th^ and 13^th^ ribs. Following postpartum palpation, cows were assessed for metritis, characterised by foetid, watery, red-brown discharge within 21 d of partition. Puerperal metritis was diagnosed in cows with rectal temperatures exceeding 39.5°C. A pain reaction indicative of inflamed or impaired hoof function was diagnostic for lameness. A retained placenta is the condition when a cow’s uterus fails to expel its membranes within 24 h of parturition. The frequencies of the disorders referred to above among the experimental cows are shown in Table 1.

**Table 1.** Prevalence of disease in three groups of dairy cows

Condition	Percentage affected per group		
	M (n = 40)	L (n = 52)	H (n = 64)
Ketosis	12.5 (5/40)^a^	42.3 (22/52)^b^	60.93 (39/64)^b^
Mastitis	15 (6/40)^a^	17.3 (9/52)^a^	37.5 (24/64)^b^
Retained placenta	7.5 (3/40)^a^	9.62 (5/52)^a^	34.38 (22/64)^b^
Milk fever	2.5 (1/40)	3.84 (2/52)	6.25 (4/64)
Displaced abomasum	0^a^	1.92 (1/52)	14.06 (9/64)^b^
Metritis	5 (2/40)^a^	13.46 (7/52)	25 (16/64)^b^
Lameness	10 (4/40)	15.38 (8/52)	15.63 (10/64)

1 M – maintained-BCS group; L – lost-BCS group; H – high-BCS-loss group;

a,b,c different superscript letters in any pair for the same condition indicate significant difference (P-value < 0.05)

### Statistical analysis

In the present study, cows served as the sampling unit and were treated as a random effect in all statistical models. Statistical analyses were conducted using IBM SPSS Statistics for Windows v. 19.0 software (IBM, Armonk, NY, USA). Differences in BCS loss among cows were assessed using one-way analysis of variance across the M, L and H groups. Mixed model procedures were utilised to analyse BCS and plasma metrics at –21, 0, 7, 14, 21, 28 and 50 d relative to calving. Additionally, milk-yield metrics at 7, 14, 21, 28, 50, and 90 d relative to calving were also analysed using mixed-model procedures to address correlated repeated measures. The chi-squared test was used to analyse differences in disease incidence, and pairwise comparisons were performed using the Bonferroni test.

## Results

### Body condition scores of the tested cows

**Fig. 1.**
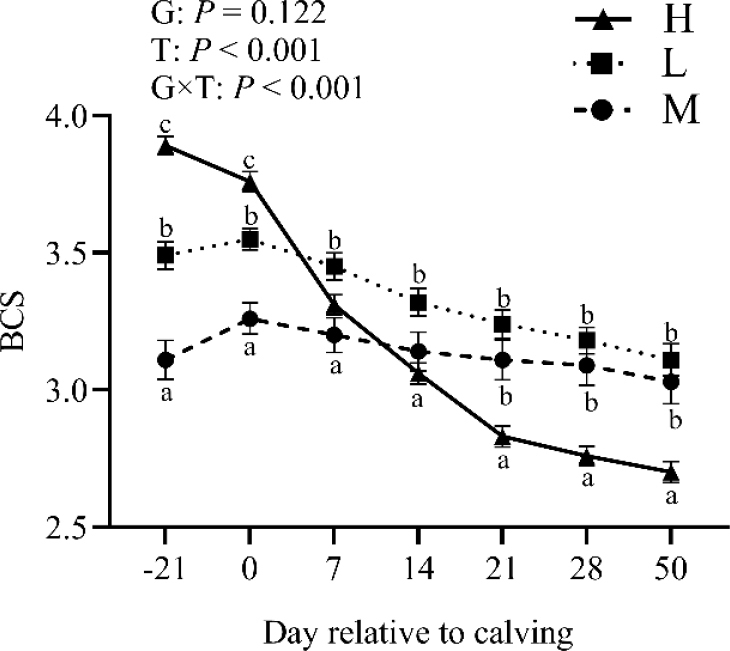
The change trend of body condition score (BCS) in the high-BCS-loss (H) group (n = 64), lost-BCS (L) group (n = 52) and maintained-BCS (M) group (n = 40) of experimental cows assessed for body condition score (BCS) a, b, c – different letters at the same time point indicate significant differences between groups (P-value < 0.05); T – time effect; G – between-group effect; G × T – inter-group interaction with time; error bars – standard error

Overall, the mean (± standard deviation – SD) BCS was 3.36 ± 0.36, with no significant rise before calving (3.49 ± 0.36 at 14 d prepartum *vs* 3.52 ± 0.31 at calving). Body condition scores progressively declined after calving, reaching their lowest point at 50 d postpartum (2.95 ± 0.41) (Fig. 1). Interestingly, no significant rise in BCS was noted in the M and L groups from 21 d prepartum to calving, when it peaked. On the other hand, a decline in BCS was noted in the H group from 21 d prepartum through 50 d postpartum. Body condition scores exhibited significant time and group × time effects (P-value < 0.01) but not a group interaction (P-value > 0.05).

### Effect of BCS loss on plasma energy metabolites

**Fig. 2.**
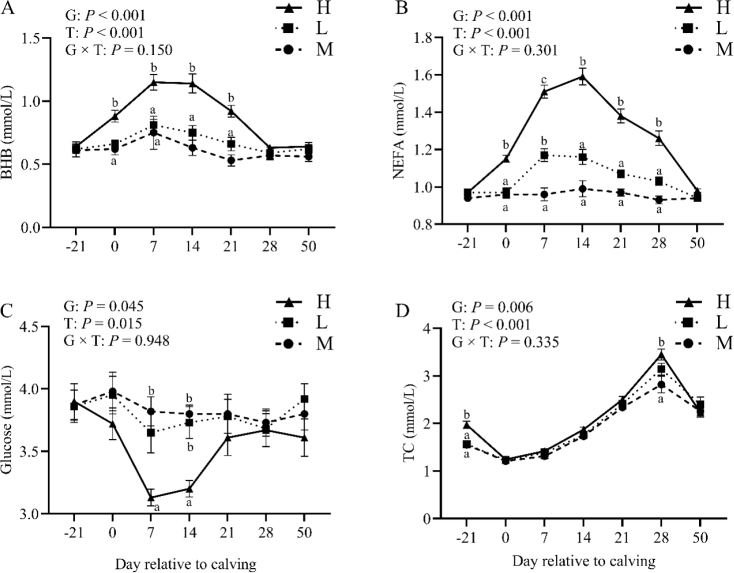
Levels of plasma β-hydroxybutyrate (BHB, A), non-esterified fatty acids (NEFA, B), glucose (C) and total cholesterol (TC, D) in the high-BCS-loss (H) group (n = 64), lost-BCS (L) group (n = 52) and maintained-BCS (M) group (n = 40) of experimental cows assessed for body condition score (BCS) a, b, c – different letters at the same time point indicate significant differences between groups (P-value < 0.05); T – time effect; G – between-group effect; G × T – inter-group interaction with time; error bars – standard error

The responses of BHB and NEFA for each group are illustrated in Fig. 2A and 2B, respectively. As anticipated, both showed similar patterns across all groups, with no significant differences between groups at 21 d prepartum. In contrast, a substantial increase was noted immediately after calving. Thereafter, BHB and NEFA steadily decreased, but the values remained higher (P-value < 0.01) in the H group compared to those in groups L and M. At 28 d postpartum, the values were comparable to prepartum levels. Beta-hydroxybutyrate and NEFA exhibited significant group and time effects (P-value < 0.01) but not a group × time interaction (P-value > 0.05).

At calving and in the prepartum period, no significant differences in glucose levels were observed (Fig. 2C). At 7 d postpartum and thereafter, glucose levels decreased. This reduction was more pronounced in the H than in the L and M groups, resulting in significant differences in glucose levels at 7 and 14 d postpartum. In contrast, differences in TC (Fig. 2D) were not apparent from calving until 21 d postpartum. However, TC levels increased after the first week of lactation. Finally, both glucose and TC exhibited significant overall group and time effects (P-value < 0.05) but no group × time interaction (P-value > 0.05).

### Effect of BCS loss on plasma liver and kidney function indicators

**Fig. 3.**
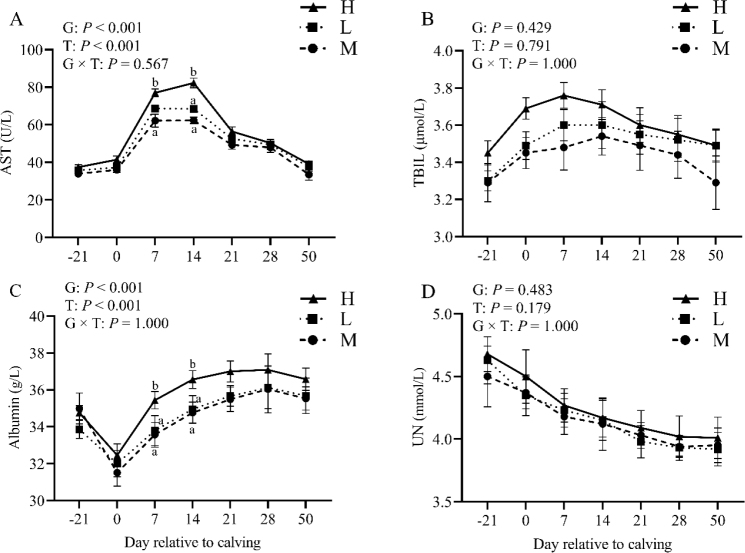
Levels of plasma aspartate aminotransferase (AST, A), total bilirubin (TBIL, B), albumin (C) and urea nitrogen (UN, D) in the high-BCS-loss (H) group (n = 64), lost-BCS (L) group (n = 52), and maintained-BCS (M) group (n = 40) of experimental cows assessed for body condition score (BCS) a, b – different letters at the same time point indicate significant differences between groups (P-value < 0.05); T – time effect; G – between-group effect; G × T – inter-group interaction with time; error bars – standard error

Group and time effects were observed in AST levels (P-value < 0.01), with group H having the highest concentration of AST (Fig. 3A) following calving. Likewise, group H had higher concentrations from 7 to 14 d postpartum, but no group × time effect was observed (P-value > 0.05). During the first week of lactation, the level of ALB increased (Fig. 3C), resulting in significant differences at 7 and 14 d postpartum. There were group and time effects for ALB (P-value < 0.01) but not a group × time interaction (P-value > 0.05). Total bilirubin and UN levels (Fig. 3B and 3D) were not comparable across group and time (P-value > 0.05). The group × time effect was not significant for these measures (P-value > 0.05).

### Effect of BCS loss on plasma oxidant stress function indicators

**Fig. 4.**
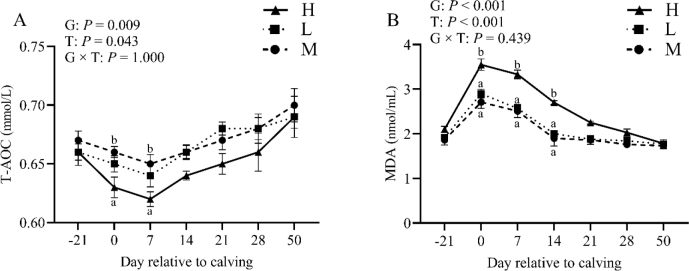
Levels of plasma total antioxidant capacity (T-AOC, A) and malondialdehyde (MDA, B) in the high-BCS-loss (H) group (n = 64), lost-BCS (L) group (n = 52), and maintained-BCS (M) group (n = 40) of experimental cows assessed for body condition score (BCS) a, b – different letters at the same time point indicate significant differences between groups (P < 0.05); T – time effect; G – between-group effect; G × T – inter-group interaction with time; error bars – standard error

Plasma T-AOC levels increased after the first week of lactation (Fig. 4A), with the values in groups L and M consistently higher than those in H (50 d postpartum). Total antioxidant capacity exhibited an overall group and time effect (P-value < 0.05) but not a group × time interaction (P-value > 0.05). Plasma MDA levels (Fig. 4B) were similar across the three groups at 21 d prepartum. As anticipated, MDA levels significantly increased before calving. Thereafter, MDA decreased. Specifically, at 28 d postpartum values were comparable to prepartum levels, and no difference was observed between groups. Group and time effects (P-value < 0.01) were observed for MDA, but not a group × time interaction (P-value > 0.05).

### Effect of BCS loss on plasma hormones

**Fig. 5.**
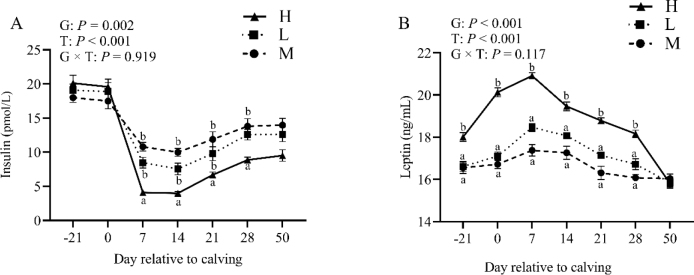
Levels of plasma insulin (A) and leptin (B) in the high-BCS-loss (H) group (n = 64), lost-BCS (L) group (n = 52), and maintained-BCS (M) group (n = 40) of experimental cows assessed for body condition score (BCS) a, b – different letters at the same time point indicate significant differences between groups (P-value < 0.05); T – time effect; G – between-group effect; G × T – inter-group interaction with time; error bars – standard error

The response of insulin for each group is displayed in Fig. 5A. Insulin levels were higher in groups M and L than in group H from 7 d postpartum through 28 d postpartum. There was an overall group and time effect for insulin (P-value < 0.01) but no group × time interaction (P-value > 0.05). Leptin levels also significantly increased after calving and subsequently decreased; for this hormone there was an overall group and time effect (P-value < 0.01) but not a group × time interaction (P-value > 0.05).

### Effect of BCS loss on plasma minerals

**Fig. 6.**
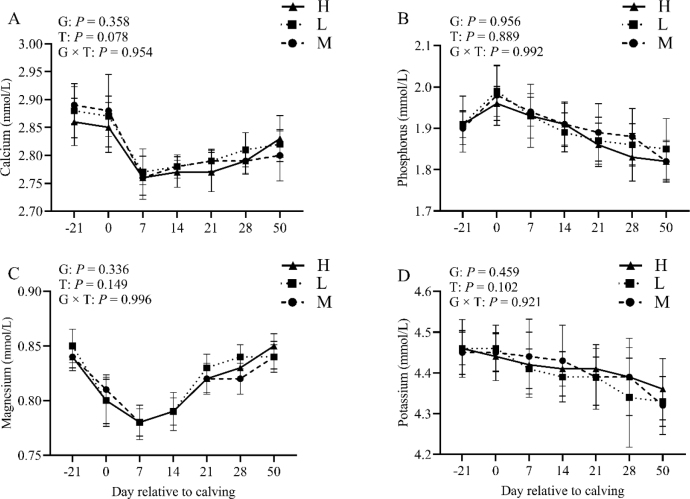
Levels of plasma calcium (A), phosphorus (B), magnesium (C), and potassium (D) in the high-BCS-loss (H) group (n = 64), lost-BCS (L) group (n = 52), and maintained-BCS (M) group (n = 40) of experimental cows assessed for body condition score (BCS) T – time effect; G – between-group effect; G × T – inter-group interaction with time; error bars – standard error

As depicted in Fig. 6, the group effect was not significant for calcium (Fig. 6A), phosphorus (Fig. 6B), magnesium (Fig. 6C) or potassium (Fig. 6D). They were not comparable across groups and time, and no group × time interaction was observed (P-value > 0.05).

### Effect of BCS loss on daily milk yield

**Fig. 7.**
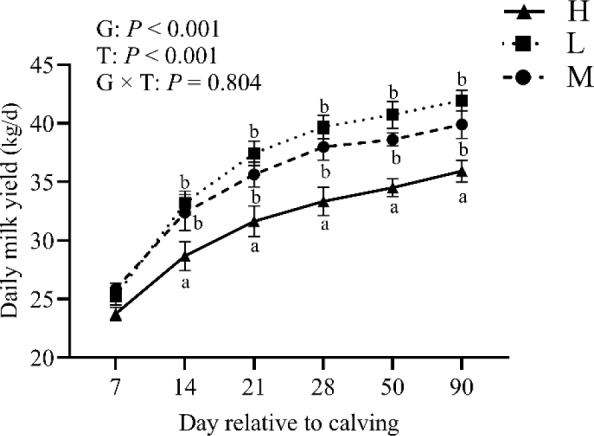
Trend of daily milk yield in the high-BCS-loss (H) group (n = 64), lost-BCS (L) group (n = 52), and maintained-BCS (M) group (n = 40) of experimental cows assessed for body condition score (BCS) a, b – different letters at the same time point indicate significant differences between groups (P-value < 0.05); T – time effect; G – between-group effect; G × T – inter-group interaction with time; error bars – standard error

Figure 7 charts the daily milk yields for the three groups. The overall mean mature equivalent milk yield was 34.24 kg/d. Cows in group L produced more milk than those in groups M and H, and significantly more than cows in group H (mean ± SD: 36.37 ± 6.98 in L *vs*. 35.06 ± 7.33 in M and 31.30 ± 7.96 kg/d in H; P-value < 0.01). There was a significant group and time effect (P-value < 0.01) but no significant group × time interaction (P-value > 0.05)

### Effect of BCS loss on disease incidence

There was no significant difference between the groups in the incidence of milk fever. Similarly, the incidence of lameness did not differ between the groups. Cows in the M group were less likely to experience ketosis, mastitis, retained placenta, displaced abomasum and metritis than those in the H group.

## Discussion

During the transition period, the mobilisation of body fat stored during the lactation stage of dairy cows results in a loss of BCS, as evidenced by physiological changes (1). Barletta *et al*. (3) described that cows with higher prepartum BCS experienced more significant BCS loss postpartum, and that cows exhibiting high BCS gradually lost BCS also prior to calving. Chebel *et al*. (7) determined that changes in BCS are closely linked to BCS during the early transition period, with fluctuations in BCS largely dependent on BCS 21 d prepartum. The experiment revealed variations in BCS at 21 d prepartum among cows with different postpartum BCS loss. Cows exhibiting greater postpartum BCS loss had demonstrated higher BCS levels at 21 d prepartum, whereas cows without postpartum BCS loss had exhibited lower prepartum BCS levels. Furthermore, cows experiencing substantial postpartum BCS loss presented with a significant decline in BCS from 21 d prepartum to parturition.

Cows affected by significant postpartum BCS loss exhibited elevated plasma levels of BHB, NEFA and TC, and decreased plasma glucose levels. Noteworthily, alterations in plasma BHB, NEFA and TC levels were most pronounced between 7 and 14 days postpartum. When there is inadequate energy intake, dairy cows undergo changes in lipid metabolism, leading to increased mobilisation of long-chain fatty acids into NEFA to sustain physiological equilibrium and triggering the utilisation of fat reserves to mitigate energy deficiencies. Adipose tissue as the primary energy reservoir in the body is particularly intensively cycled in dairy cows in early lactation, during which continuous lipolysis and fat synthesis occur, resulting in the synthesis of NEFA. During periods of excessive mobilisation of lipid reserves in dairy cows, NEFA concentrations increase, leading to the generation of acetyl-coenzyme A through NEFA metabolism. Subsequently, hepatocytes convert the excess acetyl-coenzyme A into ketone bodies via β oxidation. In instances where lipid reserves are not fully oxidised, plasma BHB levels rise. Mohsin *et al*. (24) reported that elevated fat mobilisation during early lactation in dairy cows results in decreased glucose levels and elevated NEFA and BHB levels. The elevation in plasma NEFA and TC levels in dairy cows experiencing significant BCS loss, accompanied by a reduction in plasma glucose levels, is indicative of a more severe NEB status compared to dairy cows with lesser BCS loss (32). This observation is consistent with the metabolic changes observed in the present investigation.

Additionally, cows with high BCS loss tend to exhibit higher circulating levels of AST, reflecting hepatocyte injury and its severity. Plasma AST levels have been documented to significantly rise in cases of fatty liver syndrome, hepatitis and liver cirrhosis (18). In our experiment, dairy cows with high BCS exhibited elevated plasma AST levels and a substantial increase in them between 7 and 14 d postpartum. This increase in AST levels was associated with ketosis and reduced feed intake, in line with the higher incidence of hyperketonaemia observed in the high BCS group studied by Vargová *et al*. (38). Additionally, the decrease in BCS in dairy cows was found to be correlated with ALB levels (23). The findings of this study conjointly revealed that the plasma ALB levels of dairy cows experiencing high BCS loss were higher, implying an enhanced liver protein anabolism to compensate for BCS loss. Additionally, plasma ALB concentrations decreased during labour but swiftly rebounded postpartum.

Malondialdehyde is an established marker for lipid peroxidation and oxidative stress, while T-AOC reflects the overall antioxidant status of dairy cows (33, 35). Plasma MDA concentrations in cows with varying degrees of BCS loss peaked on the day of calving and gradually decreased in the subsequent days postpartum. Cows with a high BCS and experiencing weight loss exhibited elevated levels of MDA and decreased plasma T-AOC levels. Of note is that changes in plasma MDA levels were more pronounced from the day of calving until 14 d postpartum, suggesting high perinatal oxidative stress, which, in turn, contributed to the higher serum MDA concentrations and lower T-AOC levels observed in high-BCS-loss cows compared to their counterparts. These findings indicate a compromised antioxidant capacity in dairy cows with high BCS loss and increased susceptibility to oxidative stress.

The reduction in BCS and plasma insulin levels in cows postpartum was significant and observed to be substantial by the 7^th^ d postpartum. Concurrently, cows exhibiting greater postpartum BCS loss demonstrated elevated levels of circulating NEFA, accompanied by decreased plasma glucose and insulin levels. These alterations can be ascribed to NEB (1). Insulin regulates glycaemic levels and plays a pivotal role in energy metabolism, becoming more concentrated in correlation with energy intake. Its level is influenced by NEB during the early stages of lactation. The decrease in blood glucose and insulin levels in dairy cows can be attributed to the increased demand for synthetic lactose and inadequate feed intake (47). Leptin reflects energy metabolism in dairy cows, fluctuates in level as influenced by NEB and prenatal feed intake and associates positively with BCS (20, 21). The cows exhibiting high BCS loss in the present research had stronger concentrations of leptin, which significantly increased before parturition, indicating metabolic disturbances. Previous studies have established that variations in dairy cow body condition throughout the stages of pregnancy do not impact plasma levels of calcium, phosphorus, magnesium or potassium, with which the findings of this study agree (36). More severe BCS loss in dairy cows was correlated with a higher prevalence of postpartum disorders, as well as elevated levels of NEFA and BHB. Between 7 and 14 d postpartum, NEFA and BHB concentrations were increased in circulation, leading to a higher risk of NEB in dairy cows experiencing significant BCS loss. It is worthwhile emphasizing that NEB indirectly facilitates the development of various common metabolic disorders (26). Taken together, the results from this study unveiled a significantly higher incidence of ketosis, mastitis, retained placenta, displaced abomasum and metritis in cows with substantial BCS loss compared to those with low or no BCS loss.

Cows experience inadequate feed intake and energy supply during early lactation, leading to the mobilisation of lipids to meet lactation demands. This process can potentially enhance the lactation performance of dairy cows (42). However, it is important to acknowledge that this process is a physiological regulation in dairy cows and should not be used as a means to control excessive BCS loss, as this may affect milk yields (16). In the current study, cows with varying degrees of BCS loss were monitored, with those experiencing lower BCS loss demonstrating superior lactation performance than those with higher BCS loss. This phenomenon can be associated with deeper BCS loss and NEB in dairy cows, leading to reduced feed intake and impacting lactation. Consequently, implementing appropriate management strategies to control perinatal BCS loss can enhance the lactation efficiency of dairy cows.

## Conclusion

Moderate BCS should be maintained in primiparous cows during the transition period to avoid excessive nutrient intake, which can exacerbate BCS loss, eventually culminating in NEB, liver dysfunction and oxidative stress. Therefore, appropriate management practices should be implemented to maintain stable prenatal BCS levels in primiparous cows. In addition, monitoring and adjusting BCS in high-yielding dairy cows 21 days prior to calving can potentially enhance their health and reproductive outcomes. Conversely, inadequate feeding management practices may eventually result in metabolic disorders, hormonal imbalances and stress responses in dairy cows, thereby impacting their overall health and milk production. Effective prenatal management strategies are essential to prevent overnutrition and optimise productivity in dairy cows.
